# A Novel *Col4a5*-G814fs Knock-In Mouse Model Reveals Phenotypic Heterogeneity Among Truncating *COL4A5* Mutations in X-Linked Alport Syndrome

**DOI:** 10.3390/genes17040485

**Published:** 2026-04-19

**Authors:** Yingqi Lin, Lei Sun, Mengying Li, Xinyu Kuang, Xiuli Gong, Qin Cai, Yanwen Chen, Miao Xu, Wenyan Huang, Fanyi Zeng

**Affiliations:** 1 Department of Nephrology, Rheumatology and Immunology, Shanghai Children’s Hospital, School of Medicine, Shanghai Jiao Tong University, Shanghai 200062, China; lyq01777@sjtu.edu.cn (Y.L.); sunlei@shchildren.com.cn (L.S.); imengying.li@alumni.sjtu.edu.cn (M.L.); kuangxy@shchildren.com.cn (X.K.); 2 Shanghai Institute of Medical Genetics, Shanghai Children’s Hospital, School of Medicine, Shanghai Jiao Tong University, Shanghai 200040, China; gongxl@shchildren.com.cn (X.G.); caiq@shchildren.com.cn (Q.C.); chenyw@shchildren.com.cn (Y.C.); xumiao@shchildren.com.cn (M.X.); 3 NHC Key Laboratory of Medical Embryogenesis and Developmental Molecular Biology & Shanghai Key Laboratory of Embryo and Reproduction Engineering, Shanghai 200040, China; 4 Department of Histo-Embryology, Genetics and Developmental Biology, School of Medicine, Shanghai Jiao Tong University, Shanghai 200025, China

**Keywords:** *COL4A5*, frameshift mutation, Alport syndrome, mouse model, glomerular basement membrane, genotype–phenotype correlation, lipid metabolism

## Abstract

**Background/Objectives**: X-linked Alport syndrome (XLAS) arises from pathogenic variants in *COL4A5*. Truncating variants are generally classified as severe, but whether clinically meaningful heterogeneity exists within this group remains unclear. This study aimed to establish a novel *Col4a5* knock-in mouse model based on a clinical variant and to determine whether truncating mutation position influences disease severity. **Methods**: A de novo *COL4A5* frameshift variant, c.2440delG, was identified in a patient with severe early-onset XLAS. A *Col4a5*-G814fs knock-in mouse was generated by CRISPR/Cas9 on the C57BL/6J inbred mouse strain background and compared with the established *Col4a5-G5X* nonsense model using survival analysis, serial functional measurements, kidney histopathology, transmission electron microscopy, and RNA sequencing. **Results**: The *Col4a5*-G814fs knock-in mouse was successfully generated and showed loss of glomerular α5(IV) collagen chain expression. Compared with G5X mice, G814fs mice exhibited shorter survival (median 141 vs. 161.5 days, *p* = 0.0004), earlier onset of proteinuria, and more severe kidney functional decline. By 16 weeks, G814fs mice also showed more severe glomerular basement membrane abnormalities and more extensive glomerulosclerosis. RNA sequencing revealed a shared inflammatory gene signature in both models, together with selective upregulation of genes related to the PPAR signaling pathway and fatty acid metabolism in G814fs kidneys. **Conclusions**: This study reports a novel de novo *COL4A5* frameshift variant and establishes the first *Col4a5*-G814fs knock-in mouse model. Direct comparison with the G5X model shows that distinct truncating *COL4A5* mutations can be associated with substantially different disease severity, providing a useful platform for future mechanistic and therapeutic studies in XLAS.

## 1. Introduction

Alport syndrome (AS) is a genetic kidney disease resulting from pathogenic variants in the type IV collagen genes *COL4A3*, *COL4A4*, and *COL4A5* [1]. The encoded α3, α4, and α5 chains assemble into α3α4α5(IV) heterotrimers, which constitute the major collagen IV network within the mature glomerular basement membrane (GBM). This network is essential for structural integrity and filtration barrier function [2,3]. Pathogenic variants in *COL4A5* cause X-linked AS (XLAS), the most common form of the disease, accounting for approximately 80% of cases [4]. The mutational spectrum of *COL4A5* is broad, with more than 1700 pathogenic variants catalogued in ClinVar (accessed 9 April 2026). In affected males, progression to end-stage kidney disease (ESKD) typically occurs between the second and fourth decades, depending in part on mutation type [5,6].

Large cohort studies have shown that truncating variants, including nonsense mutations, frameshifts, and large deletions, confer a significantly higher risk of early-onset ESKD than missense variants [5,7]. In current clinical practice, truncating variants are commonly grouped into a single severe prognostic category. However, whether clinically meaningful heterogeneity exists within this category remains unclear. This question is difficult to address in human cohorts because of limited sample sizes, intrafamilial phenotypic variability, and confounding by large deletions [8]. Resolving it requires experimental systems in which genetic background and environmental factors can be controlled.

Mouse models have played a key role in Alport syndrome research. Most preclinical work has relied on the *Col4a3* knockout model, which recapitulates autosomal recessive disease. This model has been used to evaluate therapies including ACE inhibitors, neutralizing IL-11 antibodies, and 4-PBA [9,10,11]. However, models of XLAS remain limited despite its higher prevalence. Only a few *Col4a5* models have been reported, the best characterized being the G5X nonsense model on the C57BL/6 background [12]. To our knowledge, no study has directly compared the phenotypic consequences of distinct truncating *COL4A5* mutations under the same genetic background, leaving an important gap in genotype–phenotype assessment for XLAS.

Here, we report a novel *COL4A5* frameshift variant, c.2440delG (p.Gly814fs), identified in a male patient who progressed to ESKD by 14 years of age. To investigate the consequences of this mid-collagenous-domain truncation, we generated an isogenic *Col4a5*-G814fs knock-in mouse on the C57BL/6J background and compared it directly with the established *Col4a5*-G5X nonsense model. Through serial phenotypic analysis and renal transcriptomic profiling, we aimed to determine whether truncating mutation position within *COL4A5* influences disease severity and to identify pathways associated with the more aggressive phenotype.

## 2. Materials and Methods

**Clinical findings and genetic analysis.** The institutional ethics board of Shanghai Children’s Hospital approved this study (No. 2020R088-E01), which followed the principles of the 2013 revision of the Declaration of Helsinki. The parents of the patient provided written informed consent. Genomic DNA extracted from the proband was analyzed by targeted next-generation sequencing covering *COL4A3*, *COL4A4*, and *COL4A5* with a SureSelect XT capture system (Agilent Technologies, Santa Clara, CA, USA) on an Illumina platform (BGI, Shenzhen, China). Candidate variants were validated in the proband and available family members through Sanger sequencing. Pathogenicity classification followed the guidelines of the American College of Medical Genetics and Genomics (ACMG).

**Generation of *Col4a5*-G814fs mice.** A single-guide RNA (sgRNA) targeting exon 30 of the murine *Col4a5* locus was synthesized (target sequence: 5′-CCAGGGCTGCCAGGAATTGG-3′). A single-stranded oligodeoxynucleotide (ssODN) donor carrying the c.2440delG modification was synthesized (5′-ACAACCTGGCCCAGTAGGGCCTCCAGGCTGCCAGGAATTGGTCTTCAGGGACCACCAGG-3′). Cas9 mRNA and sgRNA were prepared by in vitro transcription with the MEGAshortscript T7 Kit (Thermo Fisher, Waltham, MA, USA) and purified using the MEGAclear Kit (Thermo Fisher, Waltham, MA, USA). Cas9 mRNA, sgRNA, and the ssODN donor were microinjected into C57BL/6J zygotes. Injected embryos were transferred to pseudopregnant females. F0 mice were genotyped by PCR and Sanger sequencing. Correctly targeted animals were backcrossed onto C57BL/6J for at least three generations to establish stable colonies.

**Animals and experimental design.** *Col4a5*-G814fs (X^G814fs^/Y) hemizygous males were generated as described above. *Col4a5*-G5X (X^G5X^/Y) hemizygous males on the C57BL/6J background were obtained from The Jackson Laboratory (Bar Harbor, ME, USA; stock #006183). Wild-type (X^w^/Y) C57BL/6J males of the same age were sourced from Shanghai SLAC Laboratory Animal Co. (Shanghai, China) and used as controls. All mice were kept in a specific-pathogen-free environment on a 12 h light/dark schedule and given ad libitum access to standard diet and water. The institutional animal care and use committee of Shanghai Children’s Hospital reviewed and approved all procedures involving animals (No. LLSC2021076).

**Survival analysis.** X^G814fs^/Y, X^G5X^/Y, and X^w^/Y mice (*n* = 20 per group) were monitored from birth until death or a predefined humane endpoint. The observation period ended at 210 days of age. Survival was analyzed using the Kaplan–Meier method. X^G814fs^/Y and X^G5X^/Y groups were compared using the log-rank (Mantel–Cox) test. Hazard ratios and 95% confidence intervals were calculated from the survival analysis.

**Urinalysis and serum biochemistry.** Spot urine samples (20 µL) were collected in the morning (approximately 9:00 a.m.) biweekly from 8 to 16 weeks of age (*n* = 8 per group). Total urinary protein concentration was quantified using the Bradford assay (Beyotime, Shanghai, China), and urinary creatinine was measured with a creatinine assay kit (Leagene, Beijing, China). The urinary protein-to-creatinine ratio (UPCR) was then calculated. Approximately 200 µL of blood was collected at 8 and 16 weeks by retro-orbital bleeding under isoflurane anesthesia (RWD, Shenzhen, China) (*n* = 6 per group). Serum was separated, and levels of creatinine, urea nitrogen (BUN), and albumin were measured using an automatic biochemical analyzer (Hitachi, Tokyo, Japan).

**Kidney histopathology.** Mice were anesthetized with isoflurane (RWD, Shenzhen, China) and perfused transcardially with physiological saline. Kidneys were harvested from X^G814fs^/Y, X^G5X^/Y, and wild-type males at 8 and 16 weeks of age (*n* = 3 per group). Tissues were fixed in 4% paraformaldehyde overnight at 4 °C, dehydrated, embedded in paraffin, and sectioned at 4 μm thickness. Sections were stained with periodic acid–Schiff (PAS) reagent (Servicebio, Wuhan, China) to evaluate glomerular and tubular pathology. Scale bars are indicated in each panel.

**Immunofluorescence.** Kidneys were snap-frozen in OCT compound (Sakura Finetek, Torrance, CA, USA) and cut into 5 μm sections. Cryosections were fixed in acetone at room temperature for 10 min, rinsed in PBS, and incubated with an FITC-conjugated anti-type IV collagen α5 chain antibody (CosmoBio, Tokyo, Japan) at room temperature for 30 min. After washing, nuclei were stained with DAPI. Images were captured on a fluorescence microscope (Leica DM2500, Leica Microsystems, Wetzlar, Germany).

**Transmission electron microscopy.** Kidney cortical tissue was collected from a separate cohort of X^G814fs^/Y, X^G5X^/Y, and wild-type males at 8 and 16 weeks of age (*n* = 3 per group per time point). Samples were fixed in 2.5% glutaraldehyde prepared in 0.1 M phosphate buffer at pH 7.4 and 4 °C overnight, then post-fixed with 1% osmium tetroxide. After dehydration through a graded acetone series, kidney tissue was embedded in epoxy resin. Ultrathin sections (60–80 nm) were prepared on an ultramicrotome and double-stained with uranyl acetate and lead citrate. Micrographs were obtained at 12,000× magnification to assess GBM morphology and podocyte foot process architecture. Scale bars are indicated in each panel.

**RNA sequencing and bioinformatic analysis.** Whole-kidney total RNA was isolated from 16-week-old X^G814fs^/Y, X^G5X^/Y, and X^w^/Y mice (*n* = 3 per group). Poly(A)-selected mRNA libraries were prepared and sequenced on the Illumina HiSeq platform, yielding 150 bp paired-end reads. Clean reads were aligned to the mouse reference genome (mm10) with HISAT2, and transcript abundance was expressed as FPKM. Differentially expressed genes (DEGs) were called by DESeq2 (v1.46.0) running in R (v4.4.2), applying thresholds of |log2FC| > 1 and Benjamini–Hochberg-adjusted *p* < 0.05. DEGs from each mutant group relative to wild-type controls were separated by direction of change and intersected to define common and mutation-specific gene sets. Functional enrichment of GO and KEGG pathways was carried out with clusterProfiler (v4.14.6) in R, with *p*adj < 0.05 as the significance threshold. GSEA was performed on pre-ranked gene lists ordered by log2FC. Protein–protein interaction networks were assembled in the STRING database (v12.0).

**Statistical analysis.** Data are presented as mean ± SEM. For repeated functional measurements, two-way ANOVA followed by Dunnett’s post hoc test was applied. Survival curves were compared with the log-rank (Mantel–Cox) test. Statistical testing was conducted in GraphPad Prism 10.2.3, and *p* < 0.05 (two-tailed) was taken as significant.

## 3. Results

### 3.1. A Novel De Novo COL4A5 p.Gly814fs Variant in a Patient with Severe XLAS

**Figure 1:**
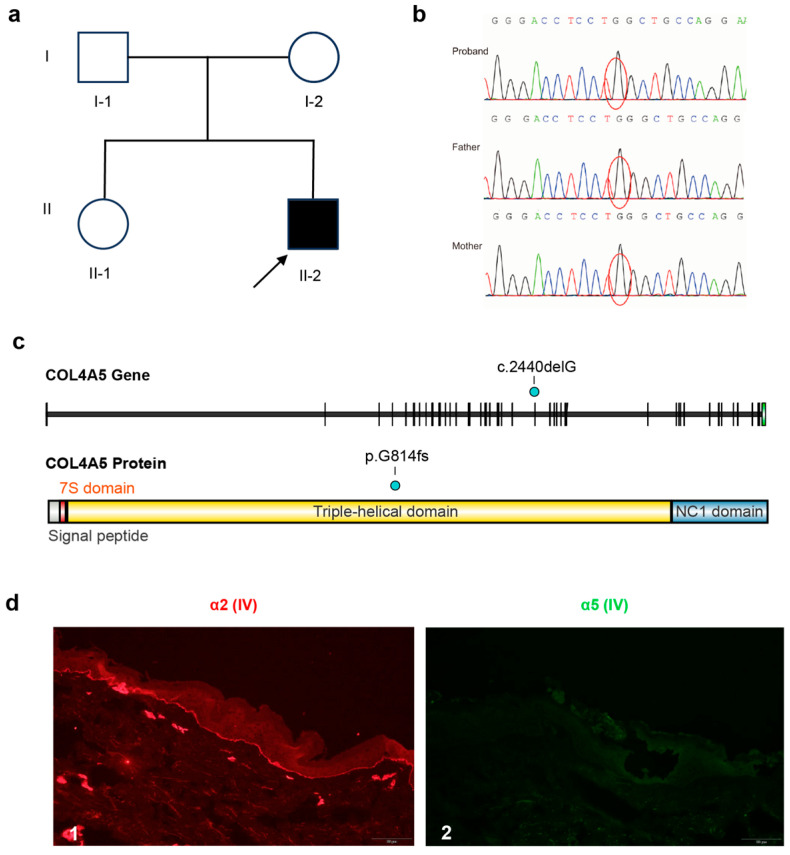
A novel de novo *COL4A5* frameshift variant identified in a patient with X-linked Alport syndrome. (**a**) Pedigree of the family. The arrow marks the proband (II-2). (**b**) Sanger sequencing chromatograms confirming the c.2440delG variant in the proband and its absence in both parents. Red circles mark the position of the c.2440delG variant. (**c**) Schematic of the *COL4A5* gene and α5(IV) protein domain structure. The cyan dot indicates the position of the c.2440delG (p.Gly814fs) variant in exon 30 within the triple-helical domain. (**d**) Immunofluorescence staining of a skin biopsy from the proband showing positive α2(IV) expression (**d1**) and negative α5(IV) expression (**d2**).

The proband (II-2) was a male who developed gross hematuria at 5 months of age following an upper respiratory infection. A kidney biopsy performed at a referring hospital at 2 years of age showed minor glomerular abnormalities with focal foot process effacement. He subsequently developed persistent proteinuria and sensorineural hearing loss. Genetic testing confirmed XLAS at 11 years of age. He progressed to ESKD at 14 years of age and received a kidney transplant one year later. Skin biopsy showed absent α5(IV) collagen chain expression and retained α2(IV) collagen expression (Figure 1d). His parents and sister were clinically asymptomatic (Figure 1a).

NGS of *COL4A3*, *COL4A4*, and *COL4A5* identified a hemizygous single-nucleotide deletion, c.2440delG (p.Gly814fs), in *COL4A5* (NM_033380), located in exon 30 within the collagenous domain (Figure 1c). This frameshift introduces a premature stop codon. The variant was not detected in either parent or his sister by Sanger sequencing, indicating a de novo origin (Figure 1b). The variant is absent from ClinVar and HGMD and was classified as pathogenic according to ACMG criteria.

### 3.2. Generation and Validation of Col4a5-G814fs Mice

**Figure 2:**
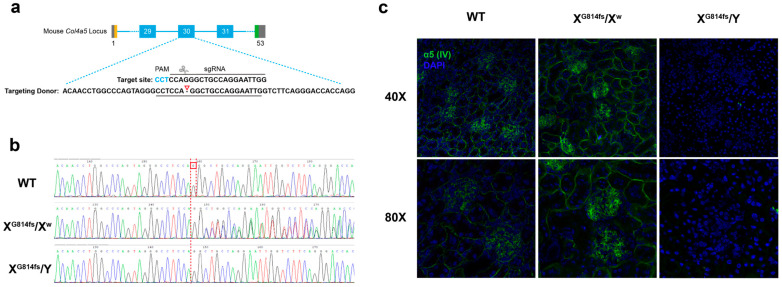
Generation and validation of *Col4a5*-G814fs mice. (**a**) Schematic of the CRISPR/Cas9 strategy targeting exon 30 of the murine *Col4a5* gene. Blue boxes indicate coding exons of *Col4a5*, with the yellow box representing the first exon and the green box representing the last exon. Gray boxes indicate untranslated regions. The PAM sequence is shown in blue. The red arrowhead indicates the Cas9 cleavage site. (**b**) Sanger sequencing chromatograms of wild-type (WT), heterozygous female (X^G814fs^/X^w^), and hemizygous male (X^G814fs^/Y) mice. The dashed line indicates the site of the single-nucleotide deletion. (**c**) Immunofluorescence staining for α5(IV) collagen (green) and DAPI (blue) in kidney sections from WT (left), X^G814fs^/X^w^ (middle), and X^G814fs^/Y (right) mice.

*Col4a5*-G814fs mice carrying the c.2440delG mutation were generated by CRISPR/Cas9 and backcrossed onto C57BL/6J (Figure 2a). Sanger sequencing confirmed correct targeting in three heterozygous females (X^G814fs^/X^w^) and three hemizygous males (X^G814fs^/Y) among 23 F0 mice (Figure 2b).

Immunofluorescence staining of the kidney showed absent α5(IV) collagen chain expression in X^G814fs^/Y mice, mosaic expression in X^G814fs^/X^w^ mice, and normal staining in wild-type controls (Figure 2c).

### 3.3. X^G814fs^/Y Mice Display a More Severe Kidney Phenotype Than X^G5X^/Y Mice

**Figure 3:**
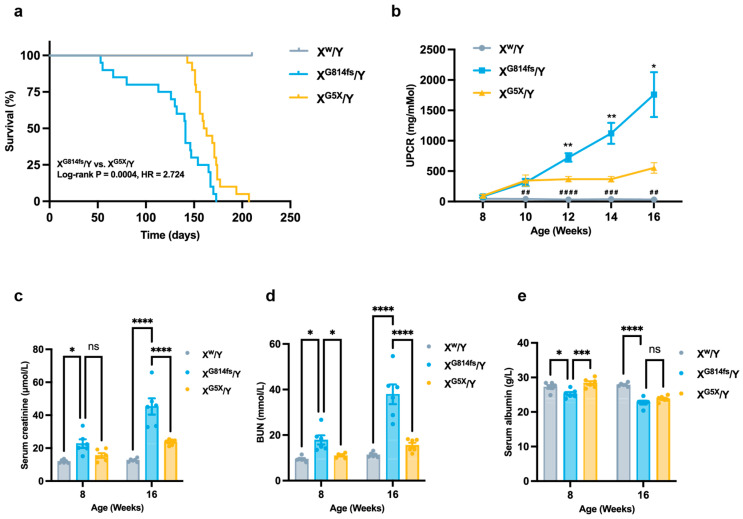
X^G814fs^/Y mice display a more severe kidney phenotype than X^G5X^/Y mice. (**a**) Kaplan–Meier curves for WT, X^G5X^/Y, and X^G814fs^/Y mice (*n* = 20). (**b**) UPCR measured biweekly from 8 to 16 weeks of age (*n* = 8). * *p* < 0.05, ** *p* < 0.01 (X^G814fs^/Y vs. X^G5X^/Y); ^##^ *p* < 0.01, ^###^ *p* < 0.001, ^####^ *p* < 0.0001 (X^G814fs^/Y vs. X^w^/Y). (**c**) Serum creatinine at 8 and 16 weeks (*n* = 6 per group). (**d**) BUN at 8 and 16 weeks (*n* = 6). (**e**) Serum albumin at 8 and 16 weeks (*n* = 6 per group). Data in (**c**–**e**) are presented as mean ± SEM. * *p* < 0.05, ** *p* < 0.01, *** *p* < 0.001, **** *p* < 0.0001; ns, not significant.

X^G814fs^/Y mice had shorter survival than X^G5X^/Y mice, with median survival times of 141 and 161.5 days, respectively. Survival analysis indicated a hazard ratio of 2.724 for X^G814fs^/Y relative to X^G5X^/Y, with a 95% confidence interval of 1.354–5.482 (*p* = 0.0004; Figure 3a).

UPCR was monitored biweekly. From 10 weeks of age, X^G814fs^/Y mice showed higher UPCR than wild-type controls (** *p* < 0.01). From 12 weeks onward, UPCR in X^G814fs^/Y mice was higher than in X^G5X^/Y mice and remained elevated through the end of follow-up (Figure 3b). At 16 weeks, serum creatinine and BUN were increased in X^G814fs^/Y mice compared with X^G5X^/Y mice (Figure 3c,d). Serum albumin was lower in both mutant groups than in wild-type controls, with no difference between X^G814fs^/Y and X^G5X^/Y mice (Figure 3e).

### 3.4. X^G814fs^/Y Mice Exhibit More Severe Histological and Ultrastructural Changes

**Figure 4:**
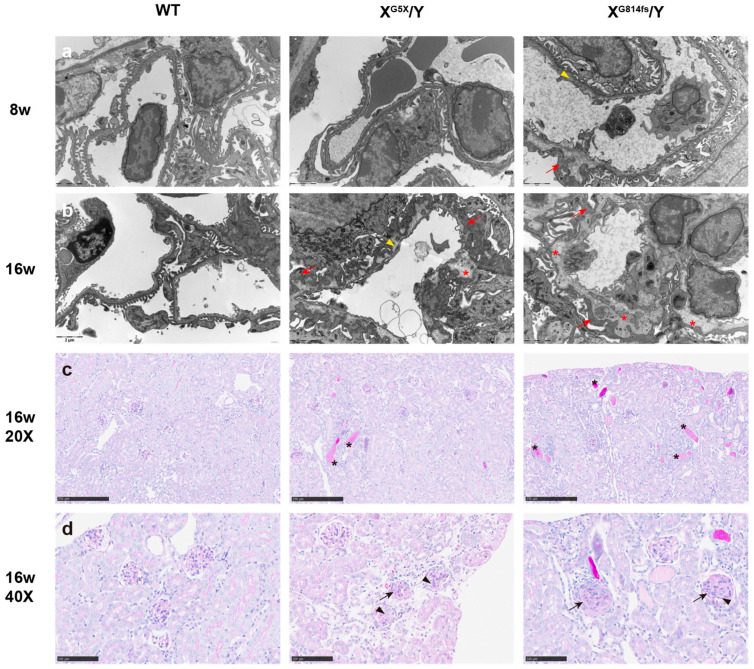
X^G814fs^/Y mice exhibit more severe histological and ultrastructural changes. (**a**) Transmission electron microscopy of glomeruli at 8 weeks. (**b**) Transmission electron microscopy of glomeruli at 16 weeks. (**c**) PAS staining of kidney sections at 16 weeks. (**d**) Higher-magnification PAS images at 16 weeks. Left, WT; middle, X^G5X^/Y; right, X^G814fs^/Y in all panels. Scale bars: 2 μm (**a**,**b**); 250 μm (**c**); 100 μm (**d**). *n* = 3 per group per time point. In electron micrographs (**a**,**b**), red arrows indicate foot process effacement; yellow arrowheads indicate GBM thickening with basket-weave transformation; red asterisks indicate splitting of the lamina densa with lamellation. In PAS-stained sections (**c**,**d**), black asterisks indicate proteinaceous casts; black arrows indicate glomerulosclerosis; black arrowheads indicate mesangial matrix expansion.

Glomerular ultrastructure was assessed at 8 and 16 weeks by transmission electron microscopy. At 8 weeks, wild-type mice displayed a GBM of uniform thickness with a well-defined trilaminar structure and regularly spaced podocyte foot processes. X^G5X^/Y mice at this time point showed normal GBM architecture and preserved foot process morphology. In contrast, X^G814fs^/Y mice already exhibited segmental foot process effacement with alternating GBM thickening and attenuation. The lamina densa contained electron-lucent areas and showed early basket-weave transformation (Figure 4a). At 16 weeks, wild-type GBM retained its normal trilaminar structure with intact foot processes. X^G5X^/Y mice developed extensive foot process effacement. The GBM showed splitting of the lamina densa with lamellation, and the normal trilaminar architecture was no longer preserved. X^G814fs^/Y mice at the same age showed widespread foot process effacement and diffuse multilaminar splitting of the lamina densa, with complete loss of the trilaminar architecture (Figure 4b).

PAS staining at 16 weeks revealed corresponding glomerular and tubulointerstitial pathology. Wild-type kidneys showed normal glomerular architecture and tubular morphology. X^G5X^/Y kidneys displayed mesangial matrix expansion, mild segmental glomerulosclerosis with partial obliteration of capillary loops, and scattered proteinaceous casts within tubular lumens. Cellular crescents were observed in occasional glomeruli. X^G814fs^/Y kidneys showed segmental-to-global glomerulosclerosis with capillary loop collapse and mesangial matrix expansion. Proteinaceous casts were more numerous and filled dilated tubular lumens. Cellular crescents were also present (Figure 4c,d).

### 3.5. RNA-Seq Identifies an X^G814fs^/Y-Specific Lipid Metabolism Signature

**Figure 5:**
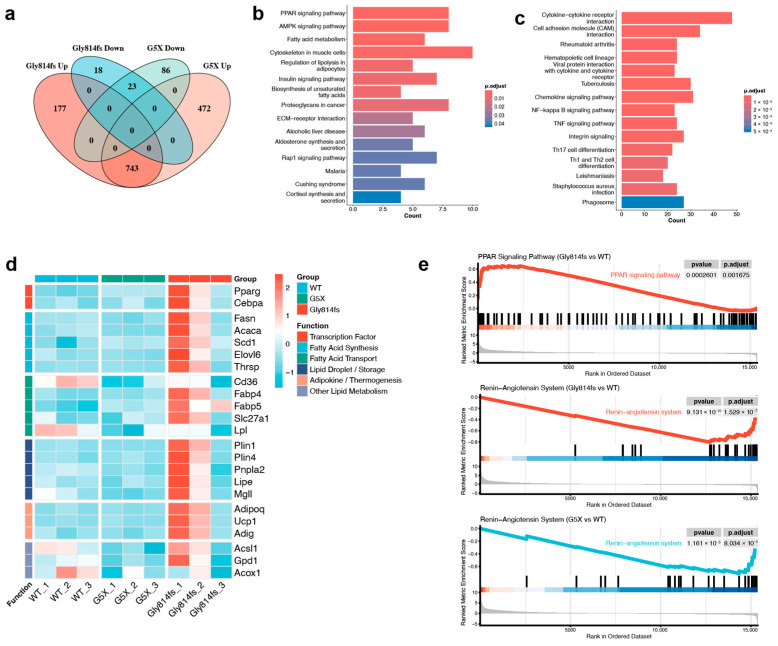
RNA sequencing identifies an X^G814fs^/Y-specific lipid metabolism signature. (**a**) Venn diagram of DEG overlap between X^G814fs^/Y and X^G5X^/Y mice relative to WT, separated by direction of change. (**b**) KEGG pathway enrichment of X^G814fs^/Y-specific upregulated genes (177). (**c**) KEGG pathway enrichment of shared upregulated genes (743). (**d**) Heatmap of G814fs-specific lipid metabolism-related core genes across WT, X^G5X^/Y, and X^G814fs^/Y kidneys. (**e**) GSEA plots. Upper panel: PPAR signaling pathway (X^G814fs^/Y vs. WT). Middle panel: renin–angiotensin system (X^G814fs^/Y vs. WT). Lower panel: renin–angiotensin system (X^G5X^/Y vs. WT). *n* = 3 per group.

Transcriptomic profiling of 16-week-old kidneys identified 961 DEGs in X^G814fs^/Y mice and 1324 DEGs in X^G5X^/Y mice relative to WT controls. Both models shared 743 upregulated genes mapping to NF-κB signaling, TNF signaling, cytokine–cytokine receptor interaction, and Th17 cell differentiation pathways (Figure 5a,c). No genes showed opposite directions of change between the two models.

A total of 177 genes were uniquely upregulated in X^G814fs^/Y mice. Enrichment analysis of this gene set identified the PPAR signaling pathway (*P*adj = 1.85 × 10^−4^), AMPK signaling pathway (*P*adj = 1.01 × 10^−3^), and fatty acid metabolism (*P*adj = 1.01 × 10^−3^) as the top enriched KEGG pathways (Figure 5b). GO analysis identified fat cell differentiation (*P*adj = 4.74 × 10^−13^) and fatty acid metabolic process (*P*adj = 4.54 × 10^−5^) as the most enriched biological processes (Supplementary Figure S1a). This gene set was also enriched for response to oxidative stress (*P*adj = 0.008), response to reactive oxygen species (*P*adj = 0.026), and response to hypoxia (*P*adj = 0.007), none of which were enriched among the shared gene set (Supplementary Table S1). Heatmap visualization confirmed selective upregulation of lipid metabolism-related genes in X^G814fs^/Y kidneys, including transcription factors (*Pparg*, *Cebpa*), fatty acid synthesis enzymes (*Fasn*, *Acaca*, *Scd1*, *Elovl6*), transporters (*Cd36*, *Fabp4*, *Fabp5*), and lipid droplet-associated proteins (*Plin1*, *Plin4*), all of which remained at baseline in X^G5X^/Y and WT kidneys (Figure 5d).

GSEA confirmed positive enrichment of the PPAR signaling pathway in X^G814fs^/Y mice (NES = +1.54, *P*adj = 1.70 × 10^−3^), whereas this pathway was not enriched in X^G5X^/Y mice (Figure 5e). The renin–angiotensin system (RAS) was suppressed in both models, with greater suppression in X^G814fs^/Y mice (NES = −2.81, *P*adj = 4.69 × 10^−8^) than in X^G5X^/Y mice (NES = −2.38, *P*adj = 3.18 × 10^−5^) (Figure 5e). PPI network analysis of the G814fs-specific genes identified a densely connected module centered on *Pparg*, with *Fasn*, *Acaca*, *Scd1*, *Elovl6*, and *Adipoq* as hub genes (Supplementary Figure S1b).

## 4. Discussion

In this study, we generated an isogenic *Col4a5*-G814fs knock-in mouse carrying a novel frameshift variant, c.2440delG, identified in a patient with severe early-onset X-linked Alport syndrome, and compared it directly with the established *Col4a5*-G5X nonsense model. Although both models lacked glomerular α5(IV) collagen chain expression, G814fs mice exhibited a consistently more severe phenotype, including shorter survival, earlier proteinuria, accelerated kidney functional decline, and more pronounced ultrastructural and histological injury. Transcriptomic profiling further revealed a shared inflammatory program in both genotypes, whereas G814fs kidneys showed additional enrichment of the PPAR signaling pathway and fatty acid metabolism-related pathways. Together, these findings support the existence of phenotypic heterogeneity among truncating *COL4A5* mutations and suggest that factors beyond the absence of α5(IV) from the GBM may contribute to differences in disease severity.

Whether the position of a truncating mutation within *COL4A5* independently influences kidney prognosis remains unresolved. A large meta-analysis identified a clear positional effect for glycine missense mutations in the collagenous domain [13], but this effect was not observed when the analysis focused on frameshift and nonsense mutations. A subsequent cohort reported worse outcomes for N-terminal variants [7], although this observation was later attributed to enrichment of large deletions in that region [8]. Our isogenic model comparison helps address this issue experimentally. Under the same C57BL/6J background and identical conditions, G814fs mice developed more severe kidney disease than G5X mice despite the absence of glomerular α5(IV) expression in both models. One possible explanation is that the two mutations differ in their effects on transcript processing and intracellular handling. The G5X mutation introduces a premature termination codon (PTC) at the fifth codon, within the signal peptide, and may therefore be efficiently subjected to nonsense-mediated mRNA decay (NMD), effectively behaving as a null allele. By contrast, the G814fs PTC lies in the mid-collagenous domain and may have a different transcript fate. Recent large-scale analyses have shown that NMD efficiency can vary according to PTC position along the transcript [14]. It is generally accepted that truncating *COL4A5* mutations abolish α345(IV) trimer assembly [15]. However, if the G814fs transcript partially escapes NMD, the resulting aberrant polypeptide could impose additional cellular stress before clearance. This possibility remains hypothetical. Direct validation would require podocyte-specific transcript quantification and protein-level analyses at early disease stages, before significant podocyte loss and cellular composition changes confound interpretation.

Computational immune cell deconvolution revealed comparable immune and stromal cell composition between G814fs and G5X kidneys (Supplementary Table S2 and Figure S2), suggesting that the phenotypic gradient between the two models is not driven by differences in immune cell infiltration. Instead, G814fs kidneys showed selective upregulation of genes converging on PPAR signaling and fatty acid metabolism. Dysregulated lipid metabolism has been implicated in kidney fibrosis and podocyte injury [16,17]. Defective fatty acid oxidation is a recognized feature of injured proximal tubular cells in fibrotic kidneys, and lipid accumulation in podocytes promotes oxidative stress and glomerulosclerosis [18,19,20]. Notably, ER stress and UPR signaling in kidney cells can directly activate lipogenic programs, and PPARγ deficiency in podocytes elevates baseline ER stress and apoptosis [21,22]. The gene expression pattern observed in G814fs kidneys is consistent with altered lipid metabolic responses associated with the more severe phenotype. Likewise, the more advanced glomerulosclerosis and tubular cast formation in these mice are compatible with accelerated tissue injury. However, the current data do not establish a causal role for lipotoxicity, and functional validation, including lipidomic analyses or pathway perturbation studies, will be needed. GSEA also revealed deeper suppression of the renin–angiotensin system in G814fs kidneys, which may indicate biological differences relevant to therapeutic responsiveness.

Preclinical research in Alport syndrome has traditionally relied on *Col4a3* knockout mice that model autosomal recessive disease, whereas models for the more prevalent XLAS form remain limited [23]. Although a few *Col4a5* mutant strains carrying nonsense or frameshift variants have been developed [12,24,25], direct comparison of distinct truncating variants on the same genetic background has not previously been reported, to our knowledge. Our study helps fill this gap. The availability of both G814fs and G5X models may also provide a useful framework for testing whether candidate therapies perform similarly across distinct truncating genotypes.

This study has several limitations. First, the transcriptomic data were obtained at a single time point, 16 weeks of age. Longitudinal analyses at earlier and later time points would help clarify when the *G814fs*-associated lipid metabolic changes first emerge and how they evolve. Second, the PPAR-centered lipid signature requires functional validation, such as lipidomic profiling or in vivo pathway modulation, to assess its biological and therapeutic relevance, and systemic metabolic phenotyping would help determine whether the observed lipid metabolic changes are confined to the kidney or reflect a broader metabolic disturbance. Third, although the use of a single inbred background ensures that observed differences are attributable to the mutations themselves, testing the same mutations on alternative genetic backgrounds would further assess the generalizability of these findings. Finally, although our models isolate the effect of mutation position under controlled experimental conditions, confirmation of these positional effects in human disease will require re-evaluation of large, well-annotated clinical cohorts.

## 5. Conclusions

In conclusion, direct comparison of two isogenic *Col4a5* mutant mouse models showed that the two truncating mutations studied can be associated with markedly different disease severity. The more aggressive phenotype of G814fs mice was accompanied by a distinct renal transcriptomic profile enriched for PPAR signaling and fatty acid metabolism-related pathways. These findings refine current genotype–phenotype understanding in XLAS and provide a useful preclinical platform for future mechanistic and therapeutic studies.

## Data Availability

The RNA sequencing data presented in this study have been deposited in the NCBI Sequence Read Archive (SRA) under BioProject accession number PRJNA1447127. Additional data are available from the corresponding authors upon reasonable request.

## Supplementary Materials

The following supporting information can be downloaded at: https://www.mdpi.com/article/10.3390/genes17040485/s1, Figure S1: Additional transcriptomic analyses of X^G814fs^/Y-specific genes. (a) GO biological process terms enriched among the 177 genes upregulated only in X^G814fs^/Y. (b) Protein–protein interaction (PPI) network of G814fs-specific genes constructed using the STRING database. Figure S2: MCPcounter immune cell deconvolution barplot showing abundance scores for nine immune and stromal cell populations across WT, X^G5X^/Y, and X^G814fs^/Y kidneys. Table S1: Complete GO biological process enrichment results for the 177 genes uniquely upregulated in X^G814fs^/Y kidneys. Table S2: MCPcounter immune cell deconvolution statistics.

## Abbreviations

The following abbreviations are used in this manuscript:

AS	Alport syndrome
XLAS	X-linked Alport syndrome
GBM	glomerular basement membrane
ESKD	end-stage kidney disease
NGS	next-generation sequencing
sgRNA	single-guide RNA
ssODN	single-stranded oligodeoxynucleotide
UPCR	urinary protein-to-creatinine ratio
BUN	blood urea nitrogen
PAS	periodic acid–Schiff
DEG	differentially expressed gene
GSEA	gene set enrichment analysis
PPI	protein–protein interaction
PTC	premature termination codon
NMD	nonsense-mediated mRNA decay
RAS	renin-angiotensin system
HGMD	Human Gene Mutation Database
