# Supplementary figures and images for: A Novel Col4a5-G814fs Knock-In Mouse Model Reveals Phenotypic Heterogeneity Among Truncating COL4A5 Mutations in X-Linked Alport Syndrome
Source: Genes (Basel). 2026 Apr 19;17(4):485. doi: 10.3390/genes17040485 (PMC13115719; doi:10.3390/genes17040485)

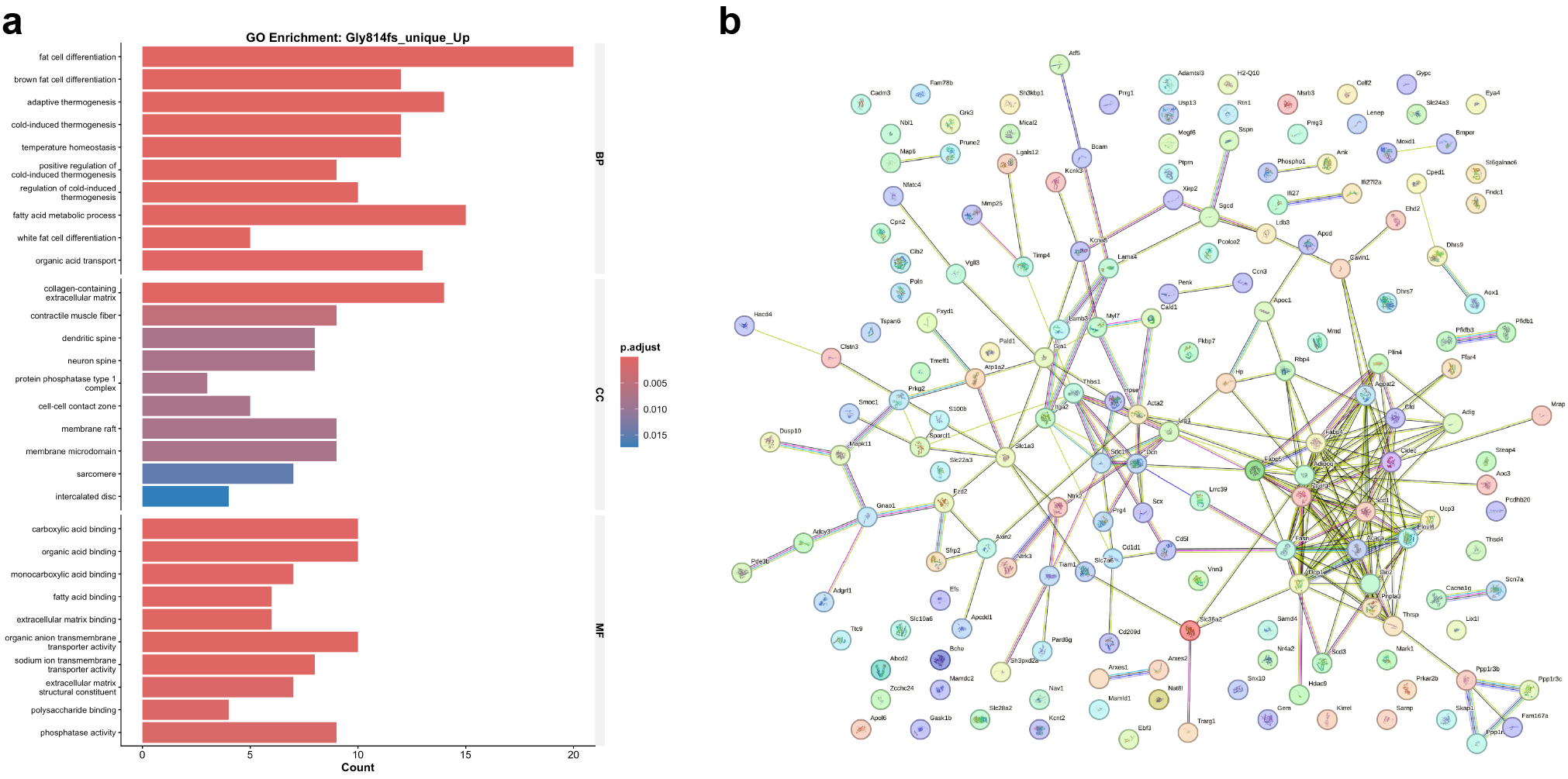

Supplement: Supplementary file 1 [file genes-17-00485-s001.zip › Supplementary Figure S1.png]

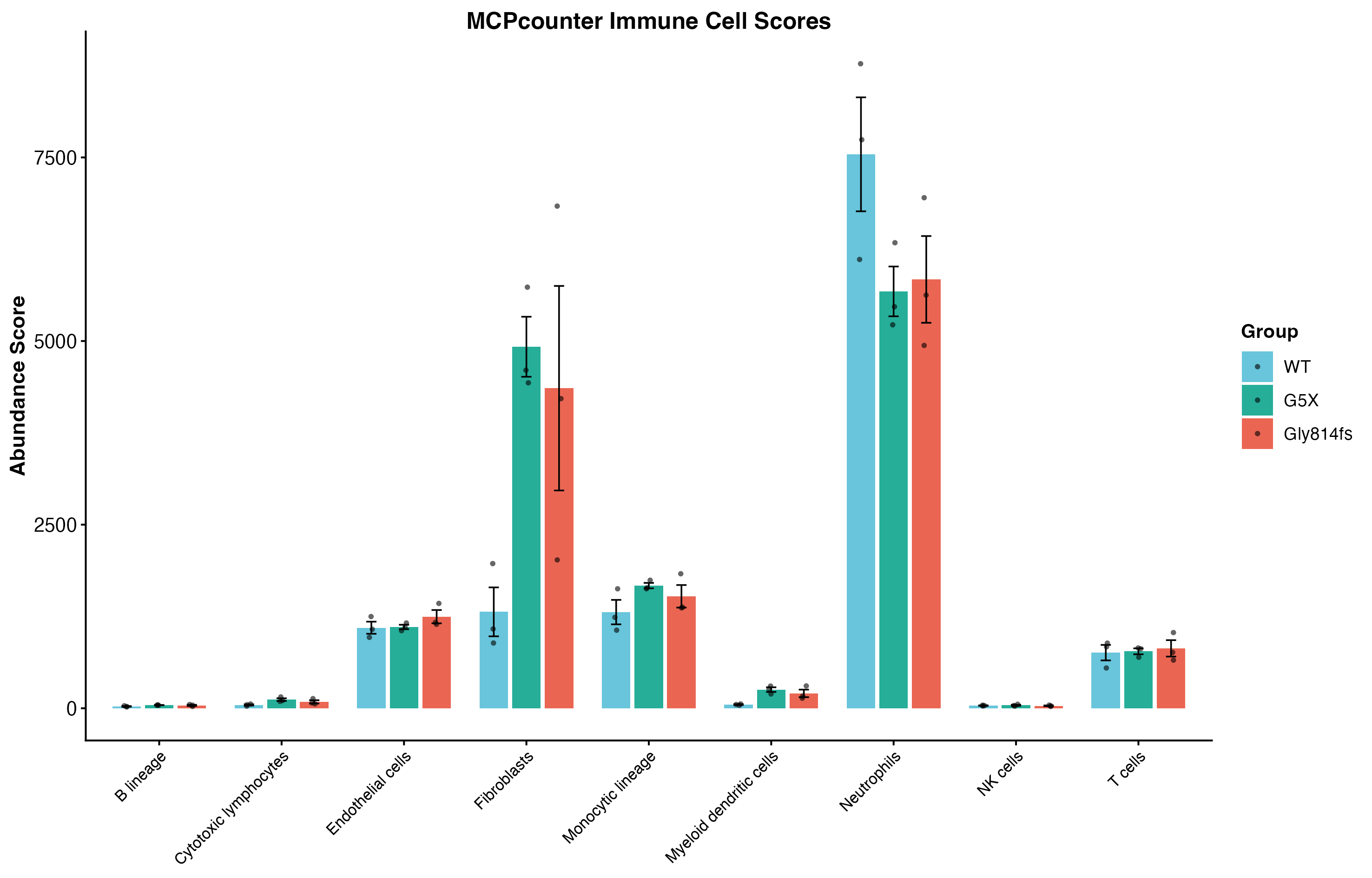

Supplement: Supplementary file 1 [file genes-17-00485-s001.zip › Supplementary Figure S2.tiff]
